# Development and validation of an autophagy-related long non-coding RNA prognostic signature for cervical squamous cell carcinoma and endocervical adenocarcinoma

**DOI:** 10.3389/fonc.2022.1049773

**Published:** 2022-11-03

**Authors:** Shuguang Zhou, Weiyu Zhang, Wujun Cao, Qinqin Jin, Xiya Jiang, Xiaomin Jiang, Yinting Yang, Hui Yao, Guo Chen, Wei Gao, Yuting Zhu, Jian Qi, Zhuting Tong

**Affiliations:** ^1^ Department of Gynecology, Anhui Medical University Affiliated Maternity and Child Healthcare Hospital, Hefei, China; ^2^ Department of Gynecology, Anhui Province Maternity and Child Healthcare Hospital, Hefei, China; ^3^ Department of Clinical Laboratory, Anhui Province Maternity and Child Healthcare Hospital, Hefei, China; ^4^ Anhui Province Key Laboratory of Medical Physics and Technology, Institute of Health and Medical Technology, Hefei Institutes of Physical Science, Chinese Academy of Sciences, Hefei, China; ^5^ Department of Radiation Oncology, The First Affiliated Hospital of Anhui Medical University, Hefei, China

**Keywords:** cervical squamous cell carcinoma and endocervical adenocarcinoma (CESC), long noncoding RNA (lncRNA), autophagy-related genes (ARGs), autophagy-related lncRNAs (ARLs), prognostic signature, immune infiltration

## Abstract

**Background:**

In this study, we aimed to investigate the signature of the autophagy-related lncRNAs (ARLs) and perform integrated analysis with immune infiltration in cervical squamous cell carcinoma and endocervical adenocarcinoma (CESC).

**Methods and results:**

The UCSC Xena and HADb databases provided the corresponding data. The ARLs were selected *via* constructing a co-expression network of autophagy-related genes (ARGs) and lncRNAs. Univariate Cox regression analysis combined with LASSO regression and multivariate Cox regression analysis were utilized to screen lncRNAs. The ARL risk signature was established by Cox regression and tested if it was an independent element bound up with patient prognosis. We used the xCell algorithm and ssGSEA to clarify the pertinence between immune infiltration and the expression of ARLs. Finally, we predicted the sensitivity of drug treatment as well as the immune response. Results indicated that the three prognostic ARLs (*SMURF2P1*, *MIR9-3HG*, and *AC005332.4*) possessed significant diversity and constituted the ARL signature. Risk score was an individual element (HR = 2.82, 95% CI = 1.87–4.30; *p* < 0.001). Immune infiltration analysis revealed significant increases in central memory CD8^+^ T cells, endothelial cells, CD8^+^ naive T cells, and preadipocytes in the high-risk group (*p* < 0.05). There were 10 therapeutic agents that varied significantly in their estimated half-maximal inhibitory concentrations in the two groups. According to the experimental validation, we found that *SMURF2P1* belongs to the co-stimulatory genes and might assume greater importance in the development of cervical adenocarcinoma. *MIR9-3HG* and *AC005332.4* belonged to the tumor-suppressor genes and they may play a more positive role in cervical squamous cell carcinoma.

**Conclusions:**

This research explored and validated a novel signature of the ARLs, which can be applied to forecast the prognosis of patients with CESC and is closely associated with immune infiltration.

## Introduction

Cervical squamous cell carcinoma and endocervical adenocarcinoma (CESC) has become one of the world’s leading gynecological cancers. It ranks 14th among all tumors and is the fourth-ranked tumor among gynecological tumor diseases all over the world. Even though there are some advances in the integrated therapy of cervical cancer, overall mortality rates increase every year around the world. At the present, CESC patients are getting younger and younger at diagnosis, and the vast majority of them have progressed to the invasive stage (1). There are limited clinical applications in squamous cell carcinoma (SCC) antigen and carbohydrate antigen 125 (CA125) because they are deficient in specificity and sensitivity (2). Hence, it is a matter of urgency to search for novel prognostic biomarkers for female patients with CESC in order to improve the prognosis, reduce the mortality rate, and develop a trustworthy, effective, and non-invasive tumor biomarker.

Long non-coding RNA (lncRNA) is one of the numerous RNAs, and most lncRNAs do not participate in the protein translation process but take part in regulating gene expression at the transcriptional or post-transcriptional level (3). There is an incremental body of facts to show that lncRNA is involved in multiple biological processes closely related to cancer (4). Studies have found that lncRNA can be expressed in the serum or plasma, which can reflect the pathophysiological changes of the patients (5). At the moment, evidence shows that lncRNA has a momentous molecular role in the germination, growth, and recurrence of tumors (6).

Autophagy was proposed after the phenomenon of “self-eating” was put forward in cells. There are massive proofs suggesting that inhibition of autophagy may be an impactful route for the treatment of advanced cancers. Research has demonstrated that genetically engineered mouse models in which autophagy-related genes (ARGs) were deleted have revealed that autophagy inhibits the growth of benign tumors but accelerates the growth of advanced cancers (7). Autophagy takes part in the pathophysiology of multiple diseases, such as metabolic diseases, infection, cancer, and so on (8). In fact, there is a controversy about the role of autophagy in cancer. New research represents that inhibited autophagy contributes to cancer development and that activated autophagy is necessary for the maintenance and development of malignant tumors (9). Various cytokines and signaling pathways, including lncRNA, can regulate autophagy processes. To date, the role of autophagy-related lncRNAs (ARLs), along with the relation between ARLs and immunity infiltration in CESC, remains obscure. Therefore, the objectives of our research were to pick out ARLs and explore their potential value in the prognostic risk assessment of CESC.

In this research, we developed a novel signature of ARLs, which has functions in prognostic prediction and potential drug selection of patients with CESC.

## Materials and methods

### Samples and data acquisition

The RNA sequencing (RNA-seq) data about CESC were from UCSC Xena (http://xena.ucsc.edu/). The expression of normalized genes was detected as a single per million mapped reads per kilobase transcript fragment and underwent log2-based transformation. The inclusion criteria were as follows: 1) patients diagnosed with CESC and 2) patients with integrated lncRNA data and clinical information. On the basis of the inclusion criteria, 309 CESC patients were incorporated. In addition to that, the TCGA database provided integrated clinical information for the patients. When filtering clinical information, samples with less than 30 days of follow-up were abandoned. Approval from the ethics committee was not required because the TCGA database supplied all clinical data related to this study and the publication guidelines of the TCGA database were strictly adhered to (http://cancergenome.nih.gov/abouttcga/policies/publicationguidelines).

### Extraction of ARGs and lncRNAs

All data for lncRNAs were obtained from RNA-seq data. The log2 transformation was used to normalize the total RNA expression data. The GENCODE (https://www.gencodegenes.org/human/release_23.html) database provided ARG information. The pertinence between lncRNAs and ARGs was determined by the Pearson correlation method. The lncRNAs relevant to autophagy were the square of correlation coefficient ∣*R* ^2^∣ >0.5 and *p <*0.001.

### Construction of the prognostic signature belonging to the ARLs

First of all, the univariate Cox regression method was used to screen ARLs. The least absolute shrinkage and selection operator (LASSO) regression was applied to test the ARLs with *p <*0.05 from the univariate analysis results. After that, the genes which were screened out by the LASSO regression were admitted to a multivariate Cox model to calculate the risk score. The risk scores were calculated as: risk scores = Ʃ (β_i_ × Exp*_i_*), in which β*_i_* indicated the weight of each signature and the Exp*_i_* indicated the expression of each gene. The patients meeting the inclusion criteria were classified into two groups according to median risk scores. The log-rank statistical test was exploited to contrast the survival difference.

### Validation of the prognostic signature

The individual prognostic signature was built to validate the prognostic features by adopting the Cox regression method. Time-dependent receiver operating characteristic (ROC) curve analysis was used to evaluate the efficacy of our signature for predicting prognostic features. These methods, which included decision curve analysis (DCA), calibration curves, and index of concordance (C-index), were applied to make a thorough inquiry into the accuracy of the signature. We included demographic data and risk scores into the multivariate Cox regression and tested if they were independent elements that were bound up with patient prognosis. We also analyzed whether the treatment outcome and pathological typing were correlated with the risk score.

### Gene set enrichment analysis

Gene set enrichment analysis (GSEA) was performed to discover the distinct enriched terms with the aim of recognizing the potential pathways, which were related to the individual prognostic signature. The pathways with *p <*0.05 and FDR <0.05 were considered statistically significant.

### Extrapolation of immune-infiltrating cells in CESC

We used the R package “xCell” and single-sample gene set enrichment analysis (ssGSEA) with the aim of quantifying the abundance of immune cells in CESC patients. The ssGSEA was achieved by the R package “GSVA,” which estimated the integrated levels of immune cell types. The xCell is an analytical approach on account of the gene signature, which integrates both the RNA-seq and microarray data and integrates the deconvolution approaches and advantages of the gene set enrichment. According to the ssGSEA and xCell instructions, gene expression profiles were prepared and the R package was run. At the same time, permutation was performed by using ssGSEA and xCell signatures.

### Prediction of the sensitivity response to therapeutic agents

The sensitivity response to therapeutic agents of CESC patients was forecasted in the light of the data derived from the Genomics of Drug Sensitivity in Cancer (GDSC; https://www.cancerrxgene.org). The half-maximal inhibitory concentration (IC_50_) was evaluated by the R package “pRRophetic.” The immune checkpoint inhibitors (ICIs) which blocked the treatment response were forecasted in the light of the Tumor Immune Dysfunction and Exclusion (TIDE) (http://tide.dfci.harvard.edu/).

### Cell lines

SiHa is a cell line of cervical squamous cell carcinoma, and Hela is a cell line of cervical adenocarcinoma. They were used as the test groups. HUCEC is a cell line of a normal cervix and is used as a negative control group. PANC-1 is a cell line of pancreatic cancer and is used as a positive control group. All of these were obtained from Shanghai FuHeng Biotechnology Company (Shanghai, China). We used DMEM containing 10% FBS (Gibco, Grand Island, NY, USA) to incubate SiHa and Hela cells. Then, we used RPMI 1640 supplemented with 10% FBS (Gibco) to incubate HUCEC and PANC-1 cells. Cells were cultured in an incubator at 37°C with 5% CO_2_.

### Quantitative real-time polymerase chain reaction

We used the TRIzol reagent (Invitrogen, Carlsbad, CA, USA) to collect and lyse the cells. Then, RNA cDNA first-strand synthesis kit (TransGen Biotech, Beijing, China) was utilized to obtain cDNA. Real-time PCR was performed with One-Step qRT-PCR Kit (Sangon Biotech, Shanghai, China), and quantitative real-time polymerase chain reaction (qRT-PCR) was carried out as follows: 95°C for 3 min and then 45 cycles of 95°C for 7 s, 57°C for 10 s, and 72°C for 15 s. The internal reference was the glyceraldehyde-3-phosphate dehydrogenase (*GAPDH*) gene. Information on the primers used in the study is provided in **Table 1**.

**Table 1:** PCR primers used in this study.

Primer name	Primer type	Primer sequence (5′→3′)
***SMURF2P1***	Forward	GACATGTCCAACCCCTGAAG
	Reverse	AGCAACCCCTCCGGACATTA
***MIR9-3HG***	Forward	TCACAGAGCAGAAGAGTGCG
	Reverse	TGTGCGGCATTACCTCTCAG
***AC005332.4***	Forward	AATGCGAGGGCACATCAAGT
	Reverse	AGAGAGAGCGAGCGAGTGTA
***GAPDH***	Forward	GGAGCGAGATCCCTCCAAAAT
	Reverse	GGCTGTTGTCATACTTCTCATGG

## Results

### Reconstruction of the co-expression network of ARGs and lncRNAs

We identified 14,234 lncRNAs in the TCGA-CESC cohort and obtained 257 ARGs from the HADb. In the ARGs, 232 genes were expressed in the TCGA-CESC cohort (**Table S1**). Moreover, a co-expression network of lncRNAs related to ARGs was constructed with the aim of identifying the ARLs. Finally, we selected 945 lncRNAs associated with autophagy (|*R* ^2^| > 0.5 and *p* < 0.001, **Table S2**).

### Appraisal of the prognostic signature relevant to ARLs

**Figure 1:**
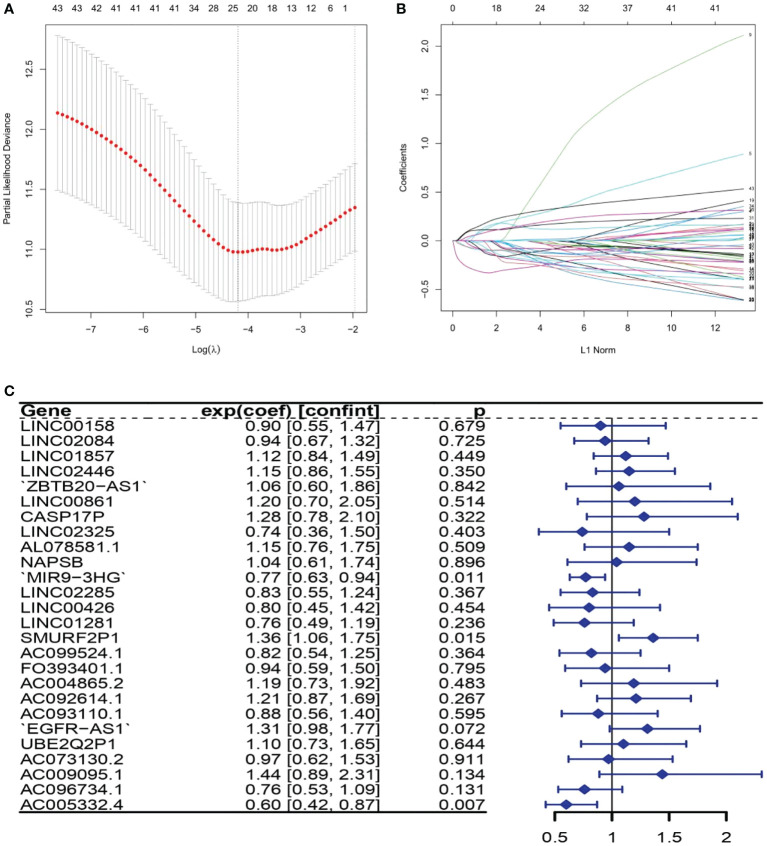
The lncRNAs related to autophagy were screened using the least absolute shrinkage and selection operator (LASSO) model: **(A)** LASSO coefficient values for 26 autophagy-related lncRNAs (ARLs) in cervical squamous cell carcinoma and endocervical adenocarcinoma (CSEC). The vertical dashed line is in the optimum log (lambda) value. **(B)** Overview of LASSO coefficients. **(C)** Forest plots show the results of the association between ARL expression and the Kaplan–Meier estimated total survival probability of CESC samples after the multivariate Cox regression analysis. Values within brackets are 95% confidence intervals of the risk ratio.

There were 43 ARLs meaningful for the patient outcome (*p* < 0.05, **Table S3**) after the univariate Cox analysis. After the LASSO regression, 26 lncRNAs associated with autophagy were filtered (**Figures 1A, B**, **Table S4**). By using multivariate Cox regression analysis, three lncRNAs were discovered to be independent prognostic indicators (**Figure 1C**). Among the three lncRNAs, there was a deleterious prognostic indicator which was named the SMAD-Specific E3 Ubiquitin Protein Ligase 2 Pseudogene 1 (*SMURF2P1*). On the other hand, the remaining MIR9-3 host gene (*MIR9-3HG*) and *AC005332.4* were beneficial prognostic indicators (**Table 2**). Therefore, we took advantage of these three lncRNAs to set up a signature of ARLs, and we calculated the risk scores as follows: Risk scores = (0.3100272 * expression value of *SMURF2P1* − 0.2640352 * expression value of *MIR9-3HG* − 0.5047942 * expression value of *AC005332.4*).

**Table 2:** The results of lncRNAs involving TCGA-CESC data after the multivariate Cox regression.

lncRNA name	Coefficient	Hazard ratio	Standard error	*Z* score	*p*-value
***MIR9-3HG***	−0.2640352	0.7679465	0.1040274	−2.538133	0.011144575
***SMURF2P1***	0.3100272	1.3634622	0.1270474	2.440249	0.014677152
***AC005332.4***	−0.5047942	0.6036298	0.1884233	−2.679043	0.007383283

### Evaluation of the prognosis by the established signature

**Figure 2:**
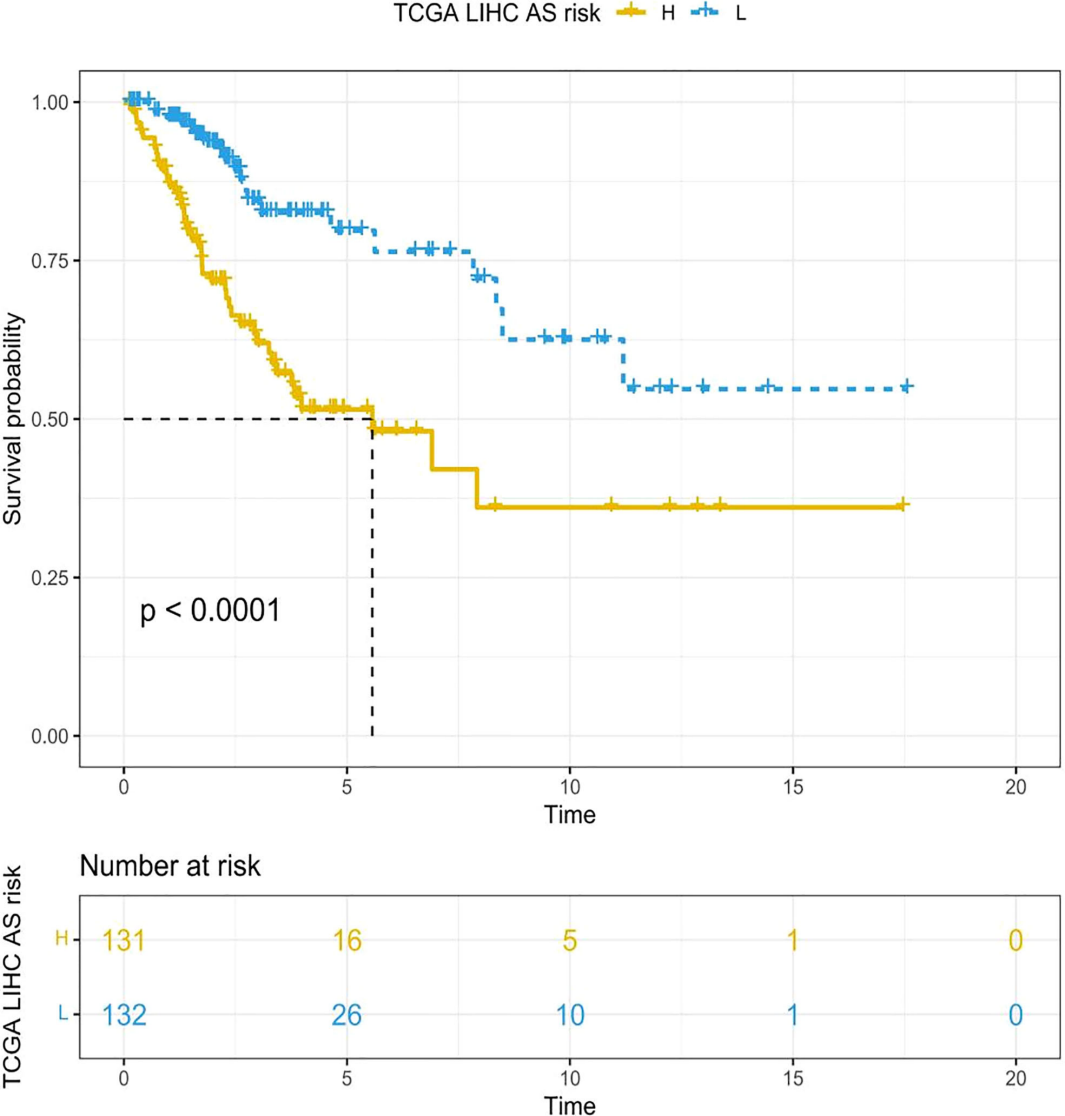
The Kaplan–Meier survival curves depicting the risk scores of the three ARLs. In comparison with the low-risk group, the group with higher risk scores had shorter OS (*p* < 0.001, log-rank test).

**Figure 3:**
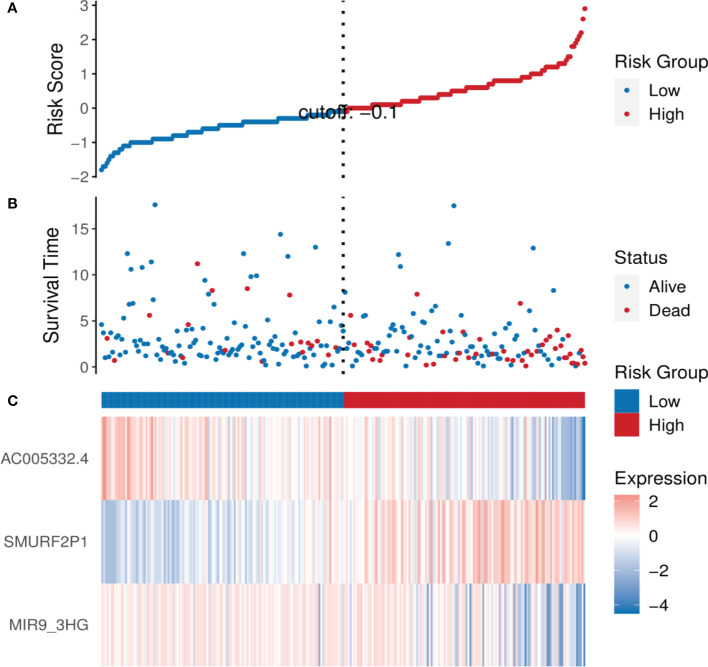
The analysis results of the ARL signature in CESC patients. **(A)** The risk scores of the two groups. **(B)** The CESC patients’ survival time. **(C)** Heatmap of the expression of the three ARLs. The upward trend from low levels to high levels respectively corresponds to the colors from green to red.

By means of the analysis of the survival curves, we could conclude that risk scores were observably relevant to overall survival (OS). In comparison with the low-risk group, the group with higher risk scores possessed shorter OS (*p* < 0.001) (**Figure 2**). Meanwhile, Cox regression results indicated that risk scores had a significant difference between the two groups, and we could see that *MIR9-3HG* and *AC005332.4* were highly expressed in the low-risk group, while the expression of *SMURF2P1* was the opposite (**Figure 3**).

### Clinical features of the signature relevant to ARLs

**Figure 4:**
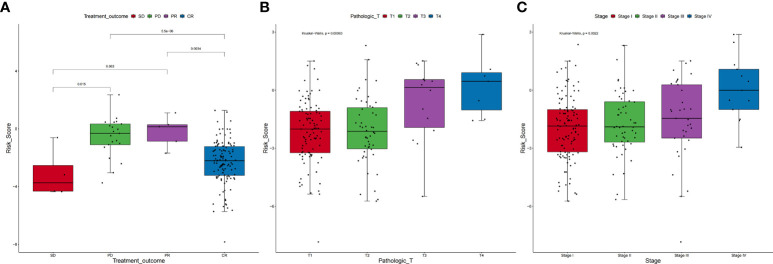
Correlation between clinical characteristics and risk scores. **(A)** Based on the analysis of the relationship between treatment outcome and risk scores, it can be seen that risk score was relatively high in the PD group and low in the SD group, but the median risk score was the highest in the PR group. Moreover, there were statistical differences between the SD and PD groups, the PD and CR groups, and the PR and CR groups. **(B)** On the relationship between Pathologic_T and risk scores, it can be seen that risk score was relatively high in T4 and low in T1, but the median risk score was the lowest in T2. **(C)** On the relationship between stage and risk scores, it can be seen that risk score was relatively high in stage IV and low in stage I, but the median risk score was the lowest in stage II.

**Figure 5:**
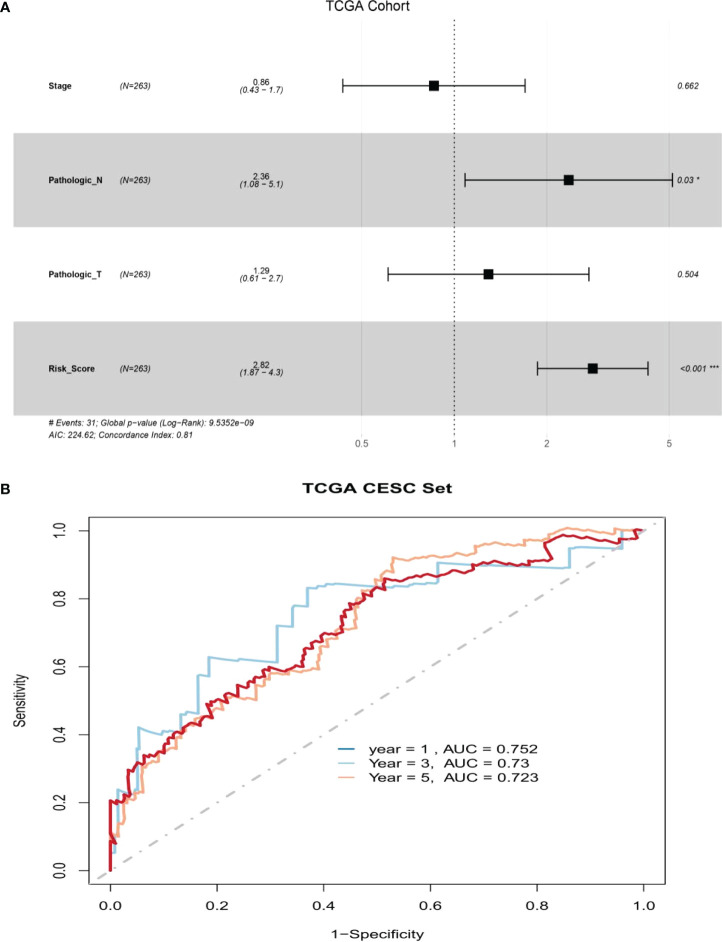
There was great predictive performance of the prognostic indicators based on ARLs. **(A)** Forest plots which represented the results of the multivariate Cox regression analysis in cervical squamous cell carcinoma. **(B)** The areas were respectively 0.752, 0.730, and 0.723, which were under the ROC curve corresponding to 1, 3, and 5 years of survival.

**Figure 6:**
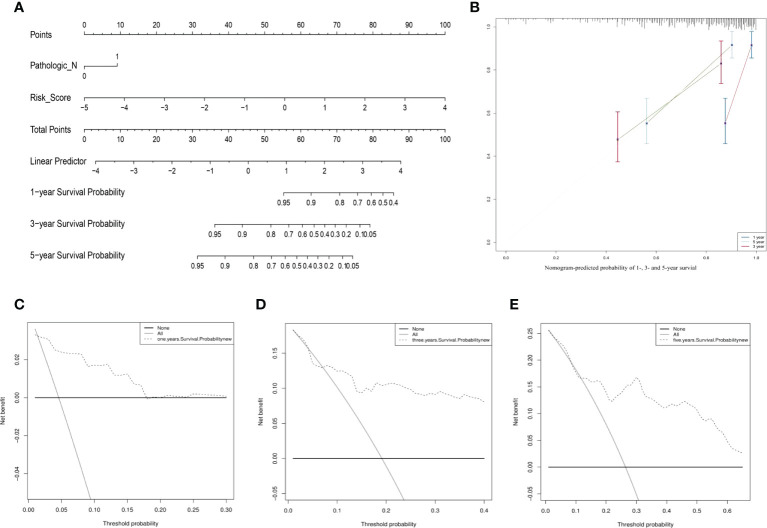
The prognostic assessment models on the basis of the three ARLs. **(A)** The nomogram depicting the 1-, 3-, and 5-year OS based on the Pathologic_N stage and risk score. **(B)** The nomogram which predicted the probability of 1-, 3-, or 5-year survival and the calibration plots which were used to estimate the consistency among the predictions of the prognostic models and the actual OS. The 45° reference line expresses ideal calibration, in which the predicted probabilities are in accordance with the realistic probabilities. The decision curve analysis (DCA) of 1- **(C)**, 3- **(D)**, and 5-year **(E)** overall survival.

The clinical features of the TCGA-CESC data are listed in **Table S5**, and the data between the two groups are listed in **Table 3**. As shown in the figures, the risk scores were statistically different in the treatment outcome, Pathologic_T, and stage. The efficacy evaluation of tumor therapy can be divided into four groups: stable disease (SD), progressive disease (PD), partial response (PR), and complete response (CR). We can see from the treatment outcome that the risk score was relatively high in the PD group and low in the SD group, but the median risk score was the highest in the PR group. Moreover, there were statistical differences between the SD and PD groups, the PD and CR groups, and the PR and CR groups (**Figure 4A**). Based on the analysis of the relationship between Pathologic_T and risk score, it can be seen that risk score was relatively high in T4 and low in T1, but the median risk score was the lowest in T2 (**Figure 4B**). When analyzing the relationship between stage and risk scores, it can be seen that risk score was relatively high in stage IV and low in stage I, but the median risk score was the lowest in stage II (**Figure 4C**). Subsequently, we also performed the multivariate Cox regression analysis using stage, Pathologic_N stage, Pathologic_T stage, and risk score as inputs. The results showed that Pathologic_N stage and risk score were robust and independent predictors of enhanced prognosis (hazard ratio: 2.82, 95% CI: 187–4.30, *p* < 0.001, **Figure 5A**). Meanwhile, the areas under the ROC curves were 0.752, 0.730, and 0.723, corresponding to the 1-, 3-, and 5-year survival rates, respectively (**Figure 5B**). Beyond these, we also made a nomogram including the risk score and Pathologic_N stage. The risk score and Pathologic_N stage had the greatest effect on OS of 1, 3, and 5 years for patients with CESC as exhibited in the nomogram (**Figure 6A**). The C-index utilized in the nomogram was 0.747 (95% CI: 0.640–0.854), which was used to predict the survival rate of OS patients (**Figure 6B**). The DCA results of the three diverse survival rates also proved that the nomogram had the potential for clinical application (**Figures 6C–E**).

**Table 3:** Clinical characteristics of TCGA-CESC data between the two groups.

	High-risk group (*n*, %)	Low-risk group (*n*, %)	*χ* ^2^	*p*
***n***	131	132		
**Age**			0.897	0.427
<60	101 (77.1)	108 (81.8)		
>=60	30 (22.9)	24 (18.2)		
**Stage**			8.917	0.030
I	61 (46.5)	82 (62.1)		
II	31 (23.6)	28 (21.2)		
III	20 (15.3)	15 (11.4)		
IV	15 (11.5)	5 (3.8)		
Unknown	4 (3.1)	2 (1.5)		
**Grade**			3.603	0.025
1	4 (3.1)	10 (7.6)		
2	55 (42.0)	65 (49.2)		
3	52 (39.7)	51 (38.6)		
4	1 (0.8)	0 (0.0)		
Unknown	19 (14.5)	6 (4.5)		
**Pathologic_T**			15.803	0.003
1	52 (39.7)	72 (54.5)		
2	31 (23.7)	32 (24.2)		
3	14 (10.7)	2 (1.5)		
4	8 (6.1)	2 (1.5)		
Unknown	26 (19.8)	24 (18.2)		
**Pathologic_M**			5.005	0.053
0	43 (32.8)	57 (43.2)		
1	8 (6.1)	2 (1.5)		
Unknown	80 (61.1)	73 (55.3)		
**Pathologic_N**			0.015	0.008
0	48 (36.6)	66 (50.0)		
1	22 (16.8)	29 (22.0)		
Unknown	61 (46.6)	37 (28.0)		
**Smoking**			0.717	0.522
No smoking	15 (11.5)	11 (8.3)		
Smoking	116 (88.5)	121 (91.7)		
**BMI**			8.880	0.012
Low weight	9 (6.9)	2 (1.5)		
Normal	35 (26.7)	27 (20.5)		
Overweight	65 (49.6)	89 (67.4)		
Unknown	22 (16.8)	14 (10.6)		

### Results of the gene set enrichment analysis

**Figure 7:**
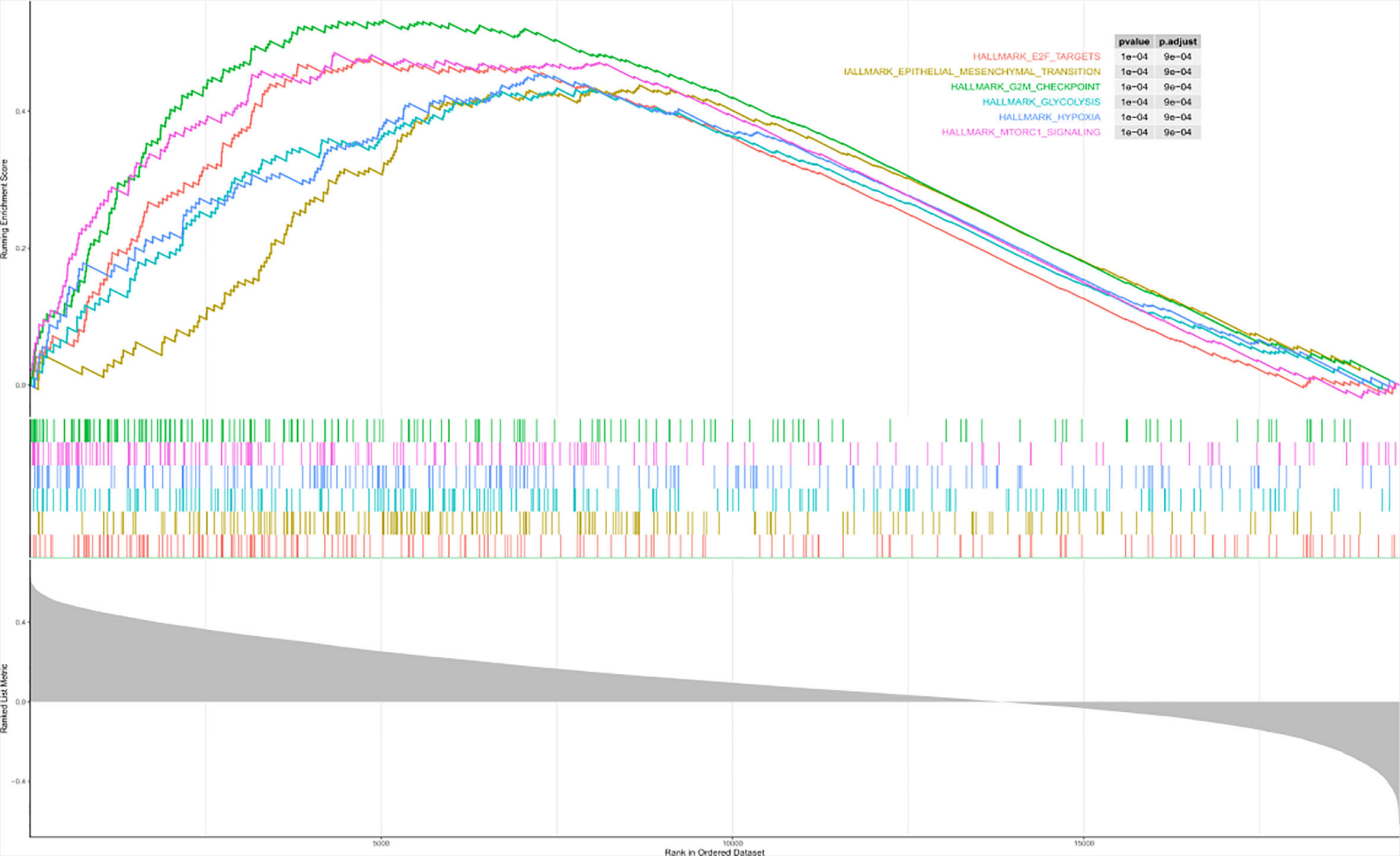
Results of the gene set enrichment analysis. There were six pathways based on the enrichment analysis, and they were respectively E2F targets, epithelial–mesenchymal transition, G2M checkpoint, glycolysis, hypoxia, and mTORC1 signaling.

With the aim of recognizing the potential pathway related to the individual prognostic signature, we performed the gene set enrichment analysis and obtained the following results (**Figure 7**). We obtained six pathways based on the enrichment analysis, and they were respectively E2F targets, epithelial–mesenchymal transition, G2M checkpoint, glycolysis, hypoxia, and mTORC1 signaling.

### Associations of ARLs with tumor-infiltrating immune cells

**Figure 8:**
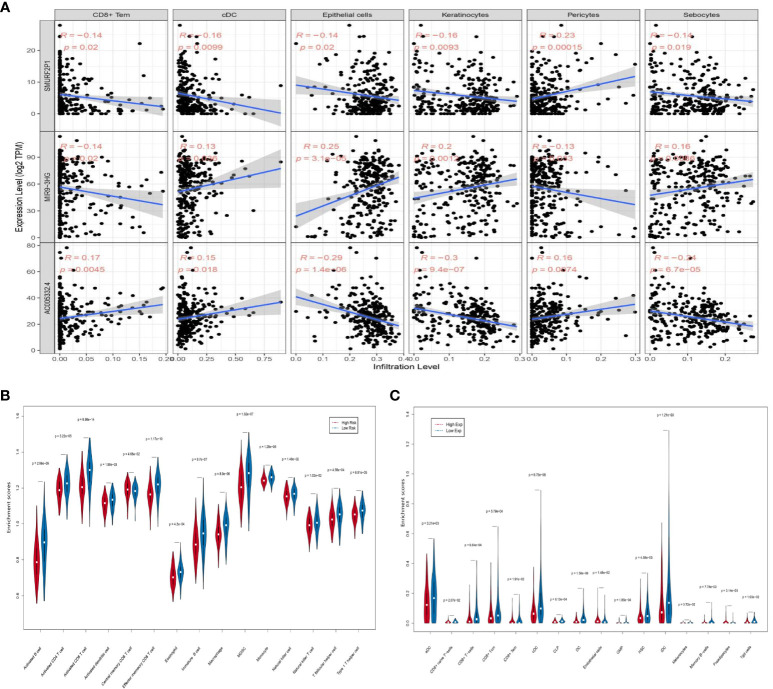
The association between the three prognostic ARLs and immune infiltration (xCell). **(A)** The pertinence between immune cell abundance in the relation to *SMURF2P1*, *MIR9-3HG*, and *AC005332.4* expression in CESC. **(B)** Violin plot which revealed that the immune cell types were differentially expressed in the two groups *via* ssGSEA. **(C)** Violin plot which revealed that the immune cell types were differentially expressed in the two groups *via* the xCell algorithm.

In our study, the comprehensive levels of the 64 immunity and stromal cell types of 263 CESC samples are listed in **Table S6**. We clarified the relevance of prognostic ARL expression (*SMURF2P1*, *MIR9-3HG*, and *AC005332.4*) and immune infiltration in CESC by using the xCell algorithm (**Figure S1**). The data demonstrated an inverse relationship between *SMURF2P1* expression and the abundance of CD8^+^ effector memory T cells (Tem) (*p* = 0.020), conventional dendritic cells (cDCs) (*p* = 0.0099), epithelial cells (*p* = 0.020), keratinocytes (*p* = 0.0093), and sebocytes (*p* = 0.019). Moreover, *SMURF2P1* expression was positively relevant to the degree of pericyte immune infiltration (*p* = 0.00015). *MIR9-3HG* expression projected a positive association with the abundance of cDCs (*p* = 0.036), keratinocytes (*p* = 0.0012), epithelial cells (*p* = 3.1e−05), and sebocytes (*p* = 0.0086), whereas the immune infiltration levels of CD8^+^ Tem (*p* = 0.020) and pericytes (*p* = 0.033) were negatively relevant to *MIR9-3HG* expression. We also discovered that the expression of *AC005332.4* was positively associated with a high number of CD8^+^ Tem (*p* = 0.0045), cDCs (*p* = 0.018), and pericytes (*p* = 0.0074) and was negatively associated with a high number of epithelial cells (*p* = 1.4e−06), keratinocytes (*p* = 9.4e−07), and sebocytes (*p* = 6.7e−05) (**Figure 8A**). This evidence suggested a significant association between ARLs screened and tumor-immune infiltration.

### Immune cell type expression between the two groups

The ssGSEA results indicated that activated B cells (*p* = 2.96e−09), activated CD4^+^ T cells (*p* = 3.22e−05), activated CD8^+^ T cells (*p* = 9.98e−14), activated dendritic cells (*p* = 1.58e−03), effector memory CD8^+^ T cells (*p* = 1.17e−10), eosinophils (*p* = 4.2e−04), immature B cells (*p* = 9.7e−07), macrophages (*p* = 8.9e−06), mDSCs (*p* = 1.32e−07), natural killer cells (*p* = 1.46e−02), natural killer T cells (*p* = 1.02e−02), and type 1 T helper cells (*p* = 6.91e−05) were negatively associated with risk score. On the other hand, central memory CD8^+^ T cells (*p* = 4.68e−02) were observably increased in the group with higher risk scores, while monocytes (*p* = 1.29e−06) and T follicular helper cells (*p* = 4.56e−04) were almost equal in the two groups (**Figure 8B**). The xCell algorithm outcome pointed out that the proportion of aDCs (*p* = 3.21e−03), CD8^+^ T cells (*p* = 9.64e−04), CD8^+^ central memory T cells (Tcm) (*p* = 5.79e−04), CD8^+^ Tem (*p* = 1.91e−02), cDCs (*p* = 6.75e−06), CLPs (*p* = 6.13e−04), DCs (*p* = 1.56e−06), GMPs (*p* = 1.85e−04), HSCs (*p* = 4.58e−03), iDCs (*p* = 1.21e−03), melanocytes (*p* = 3.72e−02), memory B cells (*p* = 7.78e−03), and Tgd cells (*p* = 1.03e−02) were negatively associated with risk score, whereas CD8^+^ naive T cells (*p* = 2.07e−02), endothelial cells (*p* = 1.48e−02), and preadipocytes (*p* = 3.14e−03) were prominently enhanced in the group with higher risk scores (**Figure 8C**).

### Prediction of the signature in response to therapeutic agents and immune checkpoint inhibitors

**Figure 9:**
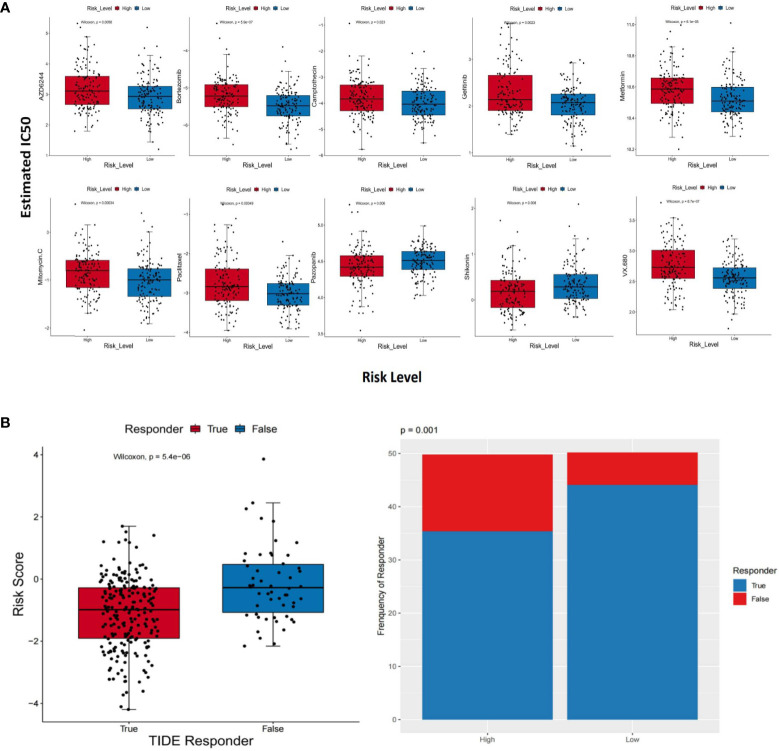
Different response sensitivities of therapeutic agents and immunotherapy. **(A)** Box plots which revealed the IC_50_ values of 10 therapeutic agents between the two groups. **(B)** The results based on the TIDE method. The risk scores between the TIDE responders and non-responders (Wilcoxon test, *p* = 5.4e−06) and distributions of responders and non-responders between the two groups (chi-square test, *p* = 0.001).

The GDSC database was utilized to obtain the IC_50_ values of therapeutic agents. Between the two groups, there were 10 therapeutic agents that varied significantly in IC_50_ values. The estimated IC_50_ values of eight therapeutic agents were higher in the group with higher risk scores, which consisted of AZD6244 (*p* = 0.0058), bortezomib (*p* = 5.9e−07), camptothecin (*p* = 0.023), gefitinib (*p* = 0.0023), metformin (*p* = 6.1e−05), mitomycin C (*p* = 0.00034), paclitaxel (*p* = 0.00049), and VX.680 (*p* = 8.7e−07). On the contrary, the estimated IC_50_ values of pazopanib (*p* = 0.006) and shikonin (*p* = 0.008) were lower in the high-risk group (**Figure 9A**). The above results implied that pazopanib and shikonin had stronger sensitivity to patients in the group with higher risk scores, and the remaining therapeutic agents showed stronger sensitivity to patients in the group with lower risk scores. Furthermore, we forecast the feasibility of reaction to immune checkpoint inhibitors using a method of the TIDE online algorithm. The risk scores were distinct between the TIDE responders and non-responders (Wilcoxon test, *p* = 5.4e−06), and the distributions of responders and non-responders were diverse between the two groups (chi-square test, *p* = 0.001; **Figure 9B**). It could be concluded that lower immunotherapy sensitivity was achieved in the high-risk group.

### Results of qRT-PCR

**Figure 10:**
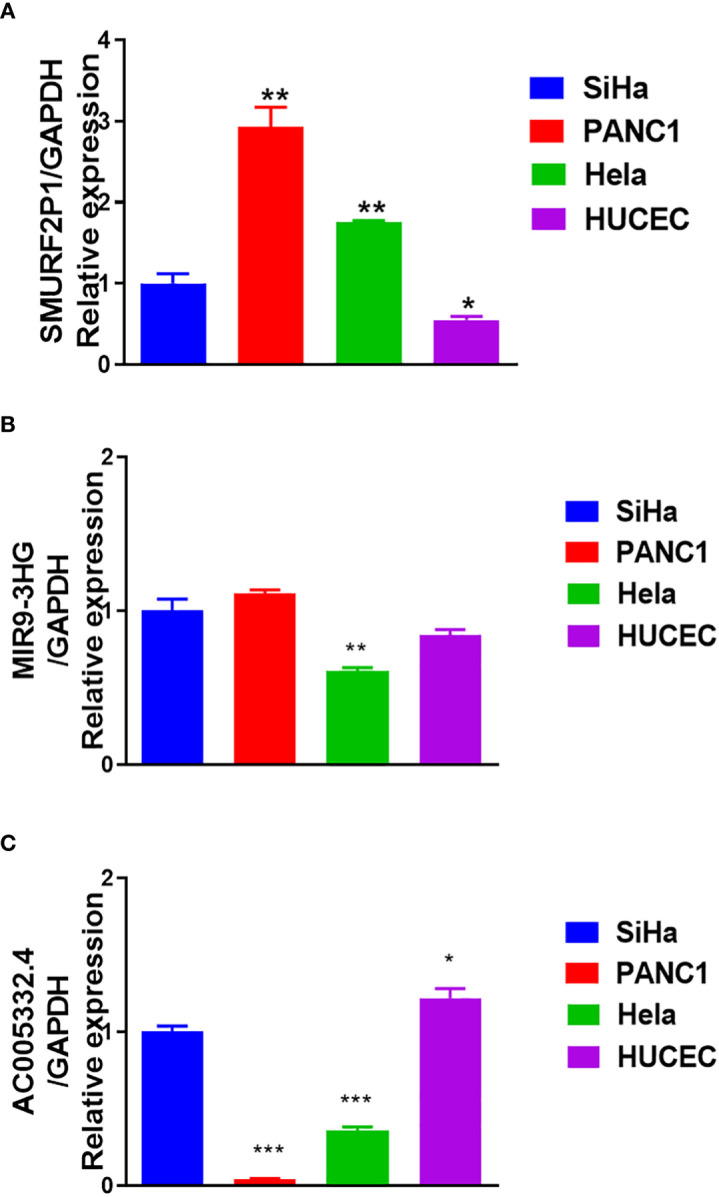
Results of quantitative real-time polymerase chain reaction (qRT-PCR). **(A)** The expression of *SMURF2P1* in the four cell lines. **(B)** The expression of *MIR9-3HG* in the four cell lines. **(C)** The expression of *AC005332.4* in the four cell lines. *p < 0.05, **p < 0.01, ***p < 0.001.

The results of qRT-PCR further revealed that *SMURF2P1* was expressed higher in Hela and SiHa cell lines compared with the HUCEC cell lines but was lower expressed in PANC-1 cell lines (**Figure 10A**). *MIR9-3HG* expression was lower in both SiHa and Hela lines than in PANC-1 cell lines. *MIR9-3HG* was also highly expressed in SiHa than in HUCEC cell lines but lowly expressed in Hela than in HUCEC cell lines (**Figure 10B**). The expression profile of *AC005332.4* was higher in SiHa cell lines than in PANC-1 and Hela cell lines but was slightly lower expressed in HUCEC cell lines (**Figure 10C**).

## Discussion

Cervical cancer could be the top untreatable disease for women with a 5-year survival rate approaching 64% (10). Admittedly, due to the lack of early non-invasive and operative detection of cervical cancer (11), it was already recurrent, persistent, or metastatic when we diagnosed it (12). Most cervical cancer patients are cured by surgery and adjuvant therapy (13). Only a small proportion of CESC patients can be cured with conventional surgery (14), while the majority of patients develop tumor recurrence and advanced metastases (15).

Autophagy is a pathway for intracellular degradation, which consists of intracellular and damaged, denatured, or aged proteins as well as the transport of organelles to lysosomes for digestion and degradation (16). An increasing number of studies have indicated that autophagy is involved in tumor progression and is related to treatment resistance (17, 18). Moreover, autophagy plays an indispensable role in cervical cancer as well (19). The effect of specific genes which take part in autophagy has become the focus of much research (20). Recently, the application of next-generation sequencing to an increasing number of cancer transcriptomes does indeed reveal that various cancer types are bound up with the unusual expression of thousands of lncRNAs (21, 22). However, few systematic studies have utilized ARL signatures which are used to forecast the survival of CESC patients.

For the sake of better understanding the function of ARLs in the germination and growth of CESC, we investigated the expression of lncRNAs in patients with CESC, which were from the TCGA database. After screening, we finally identified three genes: *SMURF2P1*, *MIR9-3HG*, and *AC005332.4*.

Next, we counted the risk scores for the three lncRNAs and established a signature of lncRNAs relevant to autophagy. Simultaneously, the patients were divided into two groups, and those with higher risk scores exhibited worse prognosis. We obtained six pathways based on the enrichment analysis, and they were respectively E2F targets, epithelial–mesenchymal transition, G2M checkpoint, glycolysis, hypoxia, and mTORC1 signaling. In the study of Xiong et al., the Rb-E2F pathway could be deemed a vital element in cervical cancer pathogenesis (23). Epithelial–mesenchymal transition was an important step in the process of cervical–epithelial carcinogenesis (24). The study of Xie et al. also showed that the 2M checkpoint pathway was associated with CESC (25). Moreover, the process of glycolysis was closely related to the proliferation and metastasis of cervical cancer cells (26). On the other hand, hypoxia was also related to the growth of cervical cancer cells (27). Meanwhile, the PI3K/AKT/mTOR signaling pathway was a vital regulatory pathway in cervical cancer (28). All of these could provide favorable evidence for our study results.

Immune infiltration is also a hot topic of research (29). It is complex and difficult to interpret the interaction between tumors and their immune microenvironment, but it is importantly implicated in the development of new prognostic markers and therapeutic strategies (30). This study clarified the relevance of the expression of lncRNAs relevant to autophagy and immune infiltration in CESC. As a result of immune cell infiltration analysis, there was an inverse correlation between risk score and aDCs, CD8^+^ T cells, CD8^+^ Tcm, CD8^+^ Tem, cDCs, CLPs, DCs, GMPs, HSCs, iDCs, melanocytes, memory B cells, Tgd cells, and so on. Furthermore, the data demonstrated an inverse relationship between *SMURF2P1* expression and the abundance of CD8^+^ Tem, cDCs, epithelial cells, keratinocytes, and sebocytes, and the immune infiltration levels of CD8^+^ Tem and pericytes were negatively relevant to *MIR9-3HG* expression. We also discovered that the expression of *AC005332.4* was positively associated with a high number of CD8^+^ Tem, cDCs, and pericytes. Meanwhile, central memory CD8^+^ T cells, endothelial cells, CD8^+^ naive T cells, and preadipocytes were increased in the group with higher risk scores. B cells have anticancer effects in human papillomavirus-associated SCC and have significant beneficial effects on patient prognosis (31). As is known to all, activated memory CD4^+^ T cells are associated with favorable outcomes in CESC patients, whereas resting memory CD4^+^ T cells are associated with adverse outcomes (32). Based on the preclinical evidence from the study of Cao et al., immunotherapy based on protein induces antitumor immune responses, which usually requires approaches based on DCs. Because DCs derived from monocyte-activated T cells restrain tumor growth by inhibiting cell propagation and accelerating apoptosis, this is influenced by the cytotoxicity of CD8^+^ T cells. Furthermore, tumor cell proliferation is inhibited by cytokines which are secreted by DCs and T cells (33). In another aspect, rapid tumor growth and lymph node metastasis are closely related to the reversion of the CD4^+^/CD8^+^ ratio and the reduced proportion of tumor-infiltrating CD4^+^ T cells in patients with cervical cancer (34). It has been shown that there were low levels of CD8^+^ Tem in cancerous tissue but high levels in lymph nodes and blood (35). Moreover, Tcm retains migration properties of naive T cells and has the ability to directly infiltrate the non-lymphoid tissues (36). These could provide a theoretical basis for our research results. In conclusion, CD8^+^ Tem may exert a positive immunological effect on CESC patients to inhibit distant tumor metastasis. *MIR9-3HG* was highly expressed in tumors, but it was a protective gene. Although the immune infiltration level of CD8^+^ Tem was negatively associated with *MIR9-3HG* expression, it still showed the protective effect of CD8^+^ Tem. However, CD8^+^ Tcm may have a role in promoting tumor metastasis.

In the light of the analysis of the therapeutic agents and immunotherapy responses to the signature we established, we could conclude that eight therapeutic agents are more treatment sensitive to the low-risk group and that immunotherapy is also more effective for patients with lower risk scores. There is a viewpoint that inhibition of autophagy can strengthen the cytotoxicity of bortezomib (37). Thus, it can be seen that the signature we established as well as the prediction of therapeutic drugs has credible evidence.

Combined with the results of qRT-PCR, we found that *SMURF2P1* belonged to the onco-stimulating gene and might have a greater role in the development of cervical adenocarcinoma. *MIR9-3HG* and *AC005332.4* belonged to the tumor-suppressor genes and they might play a more positive role in the development of cervical squamous cell carcinoma. However, in existing studies, we have not found any reports that involved the *SMURF2P1*. In some studies, *MIR9-3HG* is associated with some cancers (38–41). For example, in contrast to the normal control tissues, *MIR9-3HG* is evidently differently expressed in LIHC (41). Although *MIR9-3HG* was highly expressed in cervical cancer and could regulate apoptosis in cervical cancer cells by affecting the mitochondria-mediated apoptosis pathway (42), a study has shown that *MIR9-3HG* is a protective gene (39). Meanwhile, AC005332.4 has also been reported in colorectal cancer (43), cervical cancer (44), breast cancer (45), and osteosarcoma (46).

Overall, the autophagy-related signature that we constructed, consisting of *SMURF2P1*, *MIR9-3HG*, and *AC005332.4*, was of great significance. We obtained six pathways based on the enrichment analysis, and they were respectively E2F targets, epithelial–mesenchymal transition, G2M checkpoint, glycolysis, hypoxia, and mTORC1 signaling. The results of the response to therapeutic agents and ICIs implied that pazopanib and shikonin had stronger sensitivity to patients in the group with higher risk scores, and the remaining therapeutic agents showed stronger sensitivity to patients in the group with lower risk scores. Moreover, the results of the TIDE implied that lower immunotherapy sensitivity was achieved in the high-risk group and suggested a significant association between ARLs screened and tumor-immune infiltration.

Admittedly, our study has some limitations. First, we did not build the co-expression network which probably existed in the lncRNAs and mRNA. Moreover, experiments to demonstrate the specific molecular mechanism of ARLs (*SMURF2P1*, *MIR9-3HG*, and *AC005332.4*) in the therapy of CESC were not performed yet. We are required to make an *in-vitro* model and conduct experimental studies to further validate the assumption we proposed according to the functions of the prognostic signature of the ARLs. Therefore, the prognostic signature of the ARLs in CESC is encouraging enough to warrant advanced exploration.

## Conclusions

Based on the above data, we finally succeeded in constructing the risk score signature in the light of the three ARLs, which was an independent prognostic element in CESC patients. Our study provided profound scientific insights into the function of autophagy in the biological traits of malignant tumors. It also proposed in advance a triple ARL signature that provides effective and valuable clinic applications for dependable prognostic prediction and individuation therapy of CESC patients.

## Data availability statement

Publicly available datasets were analyzed in this study. This data can be found here: The RNA sequencing(RNA-seq) data about CESC were from UCSC Xena (http://xena.ucsc.edu/), HADb (https://www.autophagy.lu/index.html),the GENCODE (https://www.gencodegenes.org/human/release_23.html) database provided ARGs information and the sensitivity response to therapeutic agents of CESC patients was forecasted in GDSC database(https://www.cancerrxgene.org),Tumor Immune Dysfunction and Exclusion(TIDE) (http://tide.dfci.harvard.edu/) for providing their platforms and contributors for uploading their meaningful datasets.

## Author contributions

SZ, GC, ZT, and JQ conceived, designed, and supervised the study. WZ, HY, WC, QJ, XiyJ, and YY drafted the manuscript and performed the data analysis and visualization. XiaJ, WG, and YZ collected the data. All authors contributed to the article and approved the submitted version.

## Funding

This work was supported by the Applied Medicine Research Project of Hefei Health Commission (Grant No. HWKJ2019-172-14), the Research Fund Project of Anhui Medical University (Grant No. 2020xkj236), and the Natural Science Foundation of Higher Education Institutions of Anhui Province (Grant No. KJ2021A0352).

## Acknowledgments

We acknowledge the UCSC Xena, HADb, TCGA, GENCODE, GDSC, and TIDE databases for providing their platforms and the contributors for uploading their meaningful datasets.

## Conflict of interest

The authors declare that the research was conducted in the absence of any commercial or financial relationships that could be construed as a potential conflict of interest.

## Publisher’s note

All claims expressed in this article are solely those of the authors and do not necessarily represent those of their affiliated organizations, or those of the publisher, the editors and the reviewers. Any product that may be evaluated in this article, or claim that may be made by its manufacturer, is not guaranteed or endorsed by the publisher.

## Supplementary material

Supplementary Table 1 The expression profile in CESC.

Supplementary Table 2 The ARLs were screened by a co-expression network.

Supplementary Table 3 Univariate Cox results of ARLs based on UCSC Xena and HADb database.

Supplementary Table 4 Lasso regression results of autophagy-related lncRNAs.

Supplementary Table 5 TCGA CESC data in two groups.

Supplementary Table 6 The comprehensive level of 64 immunity and stromal cell types of 263 CESC samples.

Supplementary Figure 1 The relevance of prognostic autophagy-related lncRNAs expression (SMURF2P1, MIR9 - 3HG and AC005332.4) and immune infiltration in CESC by using the xCell algorithm.
