# Supplementary figures and images for: Development and validation of an autophagy-related long non-coding RNA prognostic signature for cervical squamous cell carcinoma and endocervical adenocarcinoma
Source: Front Oncol. 2022 Nov 3;12:1049773. doi: 10.3389/fonc.2022.1049773 (PMC9669765; doi:10.3389/fonc.2022.1049773)

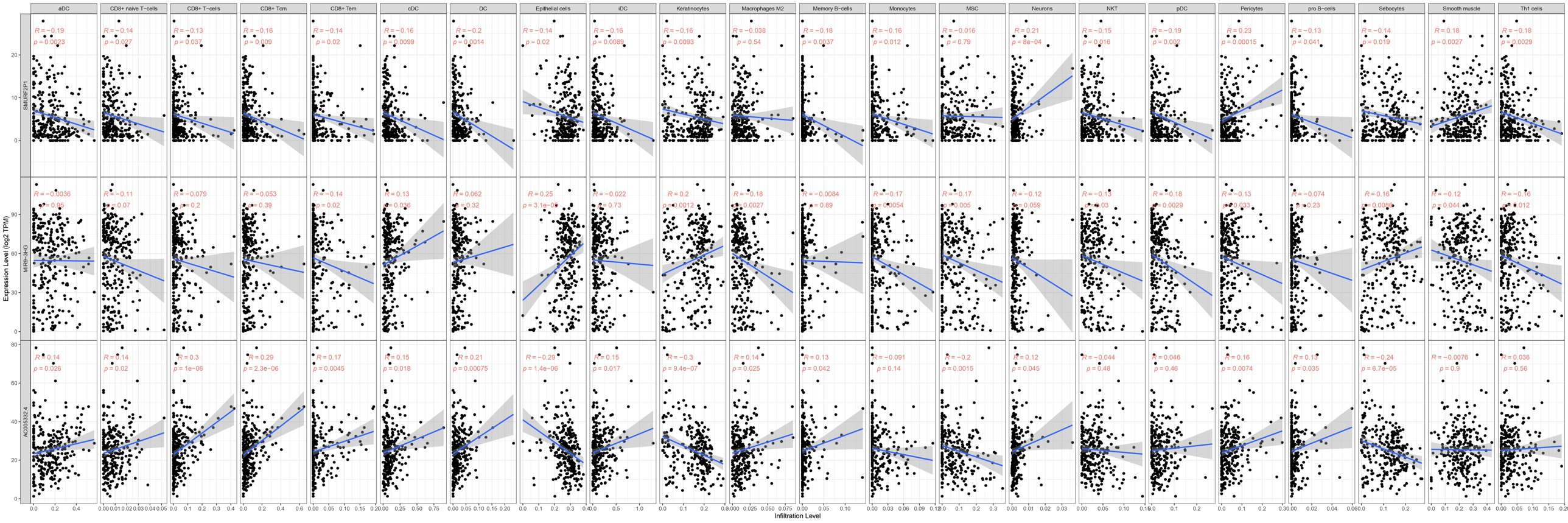

Supplement: Supplementary Figure 1 — The relevance of prognostic autophagy-related lncRNAs expression (SMURF2P1, MIR9 - 3HG and AC005332.4) and immune infiltration in CESC by using the xCell algorithm. [file Image_1.png]
